# Solution processed high aspect ratio ultra-long vertically well-aligned ZnO nano scintillators for potential X-ray imaging applications

**DOI:** 10.1038/s41598-024-61895-6

**Published:** 2024-07-09

**Authors:** Sinem V. Kurudirek, Murat Kurudirek, Anna Erickson, Nolan Hertel, Paul J. Sellin, Yauhen Tratsiak, Benjamin J. Lawrie, Charles L. Melcher, Christopher J. Summers

**Affiliations:** 1 Nuclear and Radiological Engineering, Georgia Institute of Technology, Atlanta, GA 30332 USA; 2 Department of Physics, University of Surrey, Guildford, GU2 7XH UK; 3 Department of Electricity and Energy, Technical Sciences Vocational College, Ataturk University, 25240 Erzurum, Turkey; 4 Scintillation Materials Research Center, University of Tennessee, Knoxville, TN 37996 USA; 5 Center for Nanophase Materials Sciences, Oak Ridge National Laboratory, Oak Ridge, TN 37831 USA; 6 Materials Science and Technology Division, Oak Ridge National Laboratory, Oak Ridge, TN 37831 USA; 7 Department of Nuclear Engineering, University of Tennessee, Knoxville, TN 37996 USA; 8 Department of Materials Science and Engineering, University of Tennessee, Knoxville, TN 37996 USA; 9 School of Materials Science and Engineering, Georgia Institute of Technology, Atlanta, GA 30332 USA

**Keywords:** Nanoscience and technology, Applied physics

## Abstract

We report the photon (PL), electron (CL) and X-ray (XEL) induced luminescence characteristics of high aspect ratio ultra-long (~ 50 µm) ZnO nanorods (NRs) and discuss the potential for fast X-ray detection based on the consistent and efficient visible emission (~ 580 nm) from ZnO NRs. Nanostructured ZnO scintillators were rearranged to form a vertically well-aligned NR design in order to help light absorption and coupling resulting in luminescent and fast scintillation properties. The design of the nanorod array combines the key advantages of a low-cost growth technique together with environmentally friendly and widely available materials. A low temperature hydrothermal method was adopted to grow ZnO NRs in one cycle growth and their structural, optical and X-ray scintillation properties were investigated. The relatively short (~ 10 µm) ZnO NRs emitting in the near-band-edge region were found to be almost insensitive to X-rays. On the other hand, the higher XEL response of long ZnO NRs, which is a key parameter for evaluation of materials to be used as scintillators for high quality X-ray detection and imaging, along with a decay time response in the order of ns confirmed promising scintillation properties for fast and high-resolution X-ray detector applications.

## Introduction

The increasing beneficial use of radiation in medicine, industrial, and nuclear physics applications including non-proliferation of special nuclear materials makes it crucial to develop efficient, sensitive and practical radiation detectors to detect the resulting radiation. High-resolution X- and gamma-ray detectors are required to detect radiation with both high energy resolution and high sensitivity. Commonly used X-ray detectors include solid-state detectors, gas proportional counters, and scintillators. Germanium detectors, need to be cooled to liquid nitrogen temperatures (77 K). In contrast, silicon detectors generally only have acceptable sensitivity for low energy X-rays, due to their inherent low atomic number. In gas proportional counters, the detection efficiency of X- and gamma rays is quite low, because X- and gamma rays have a very low probability of interacting when passing through a low-density gas. Among the existing detectors, scintillator detectors that can operate at room temperature have many remarkable features such as ease of use, high time resolution and high detection efficiency. NaI inorganic crystal detectors, which are among the commonly used scintillator detectors, must be kept in an airtight container to protect them from moisture due to their hygroscopic properties. Even though it has a hygroscopic nature and non-negligible lateral light spread, micro-columnar CsI(Tl) scintillators have become commonplace in many imaging detectors due to its combination of good detection efficiency and high spatial resolution^1,2^. Liquid scintillator detectors, on the other hand, are not generally useful for X-ray and gamma spectroscopy due to their lower light yield than solid scintillators and low photoelectric absorption due to their low atomic numbers^3^.

X-ray scintillation screens have been used as the core component of X-ray imaging detectors in different fields and research efforts on the development of X-ray scintillation screens were mainly focused on improving the light yield in order to enhance its sensitivity and provide high spatial resolution imaging^4–6^. There is additionally an interest in ultrafast timing performance of the X-ray imaging detectors for use in large-scale physics experimental facilities including the X-ray free electron lasers (XFELs) facility, the matter-radiation interaction in extreme (MaRIE) facility, and also for use in time of flight positron emission tomography (TOF-PET) scanners^6,7^. As a consequence, there is a need for convenient fast-timing detectors with high efficiency and resolution that operate at room temperature. Due to its properties such as ps/ns decay time, medium density and high light output, doped and undoped zinc oxide (ZnO) has the potential to be used as an inorganic scintillator in national security, nuclear non-proliferation and high energy/nuclear physics research fields^8^. When compared to the planar crystal design, the scintillation light could be better guided through the nanorods in a nano array design, which leads to less scattering but high spatial resolution. For X-ray imaging applications requiring high spatial resolution, it has been shown by simulation and experiments that vertically aligned ZnO nanoarrays could help obtain high image quality and spatial resolution^9–13^. A nanoarray scintillator, which has additional light guiding reducing the scattering of X-ray generated optical photons as described above, could play a visible-light-converting role in the visible-light-converted type 2D-detector, which will be one of the main important components to affect image resolution^14–17^. Recently, ZnO nanowire scintillators were successfully developed for high spatial resolution X-ray imaging using solution growth methods such as electrodeposition and hydrothermal^17,18^. ZnO nanorods were found to facilitate micron scale X-ray imaging which could be very useful for imaging cells and early cancer diagnosis^9^.

ZnO crystals can be obtained through different methods such as molecular beam epitaxy, radio frequency magnetron sputtering, metal-organic chemical vapor deposition, spray pyrolysis, chemical bath deposition, electrodeposition, pulsed laser deposition, laser molecular beam epitaxy, and the hydrothermal method^19–21^. Different growth techniques such as high-pressure melting technique^22^, solid-state reaction technique^23^ and high-temperature hydrothermal technique^24,25^ have been used to obtain ZnO powder or thin film that can be used as a scintillator. However, these methods generally require very high growth temperatures (800 °C–1900 °C), are expensive, result in low light output materials and require complex equipment. Among the crystal growth techniques, the hydrothermal method is a convenient method resulting in high crystallinity structures at very low growth temperatures (95 °C), and it has substrate flexibility, is low-cost, and does not require complex equipment.

While ZnO is known to emit in the UV range, this emission can be shifted to visible emission (VE) with the help of defects in the structure. Variation of growth kinetics was used to tune the VE in ZnO nanostructures for not only insight into the origin of the defect related emission but also to generate a consistent and efficient VE for visible light applications^26–32^. Huang et al.^33^ have used ZnO nanorod arrays to enhance the light extraction efficiency of a bismuth germanate (BGO) scintillator at 510 nm wavelength. Xu et al.^34^ have grown ZnO NRs with 2 µm thickness for X-ray imaging applications. Li et al.^35,36^ have developed hydrothermally grown doped ZnO micro/nano arrays to generate strong VE spectra in the range of 450–700 nm. They found that Ga and In doped ZnO micro/nano arrays exhibited a superior X-ray excited luminescence performance, which serves as a key indicator for high-spatial-resolution, fast X-ray imaging. While the VE has relatively slower decay time when compared to the UV band edge emission of ZnO, it is still in the range of a few ns, which clearly satisfies the condition for fast X-ray imaging detectors. Although they are not vertically well-aligned and inhomogeneous in shape towards the whole structure, ZnO microrods up to 100 µm emitting in the UV region were grown by a high-temperature chemical deposition technique^37^. Chen et al.^38^ have developed UV emitting ZnO nanorods of ~ 10 µm for use as an alpha particle scintillator screen. Up to now, the thickness of the ZnO nano array scintillators emitting in the VE has been reported to be up to around 20 µm. However, it is well-known that the penetration depth of X-rays in ZnO is much more than the µm scale as can be calculated using the available data^39^. Therefore, ZnO nano arrays of longer length will increase the X-ray interaction probability, increasing energy transfer to the interacting medium thereby enhancing scintillation output. Studies including the VE in ultra-long ZnO nano arrays to be used as an X-ray scintillator have yet to be done. In the present work, we have followed the low temperature hydrothermal method to grow vertically aligned ultra-long (~ 50 µm) ZnO nano arrays in one cycle growth and have investigated their structural, optical and X-ray scintillation properties.

## Results and discussion

**Figure 1:**
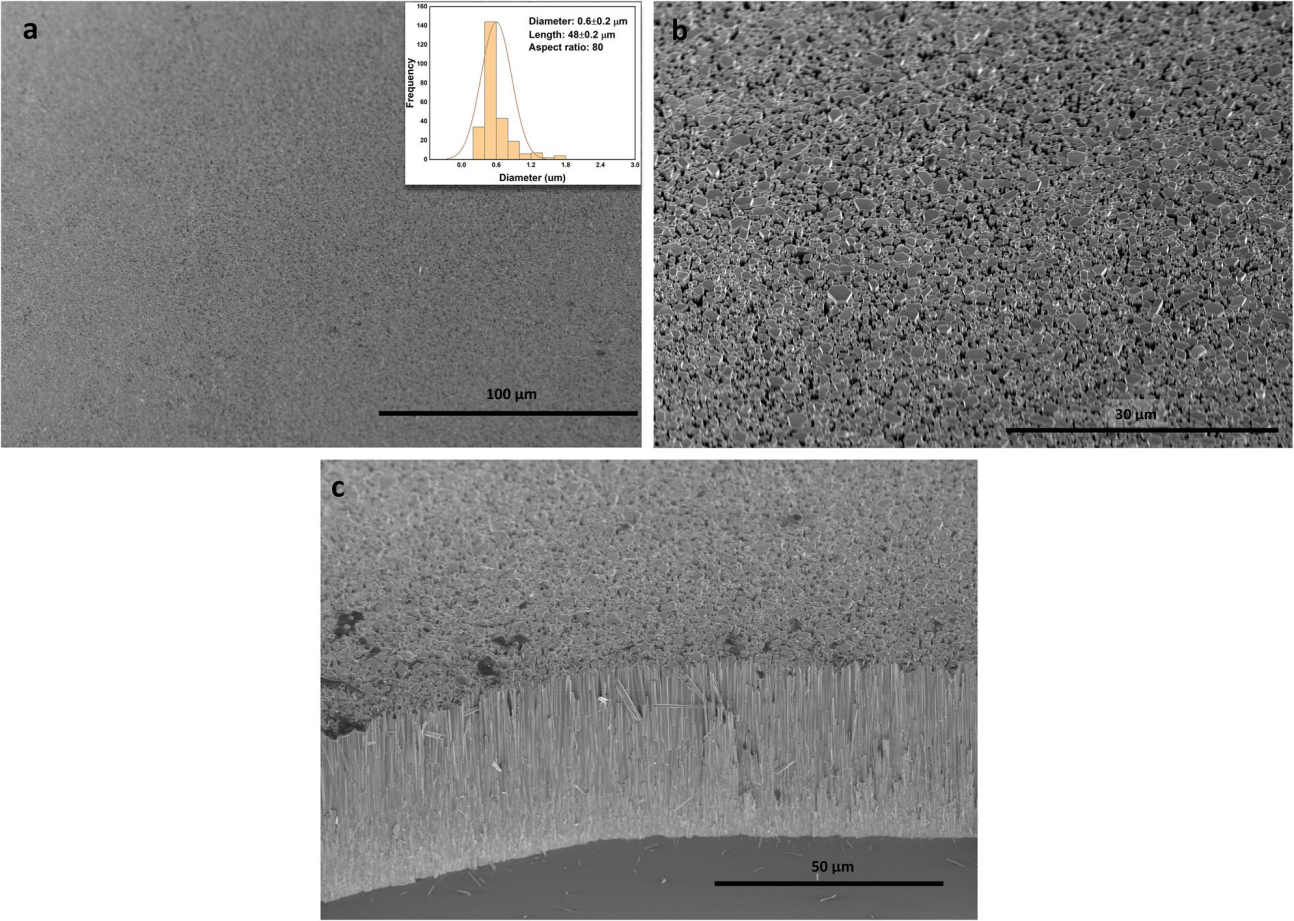
SEM images of the grown ZnO NRs (**a**) low magnification top view image, NR diameter vs. frequency (inset) (**b**) high magnification top view image (**c**) cross sectional view of ZnO NRs.

**Figure 2:**
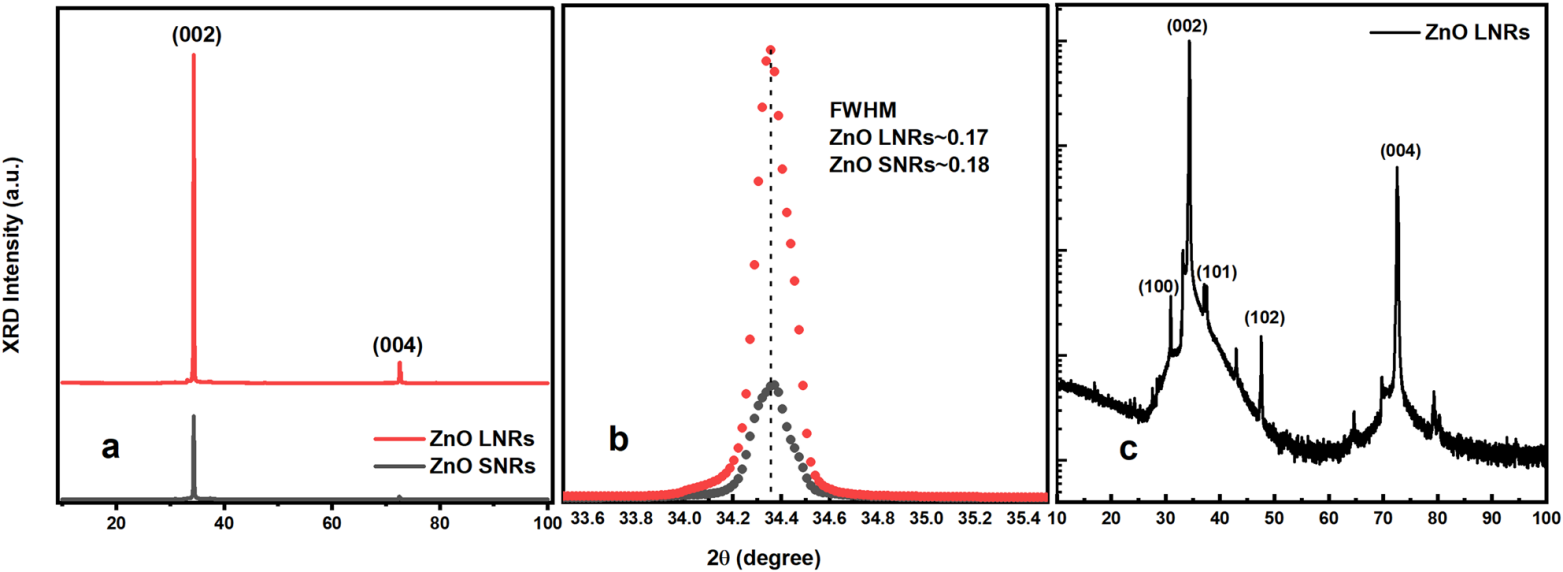
XRD patterns confirming hexagonal wurtzite structure of the ZnO NRs which grow preferentially along c-axis (**a**) sharp and intense peak at 002 crystallographic plane (**b**) magnified peaks (002) with FWHM data (**c**) semi-log plot indicating weak diffraction peaks along with dominant (002) peak.

Typical SEM images of the synthesized ZnO NRs are shown in Fig. 1. Very dense and homogeneously distributed ZnO NRs with hexagonal facets were obtained. Figure 1a and b represent the low and high magnification images, respectively. Figure 1a confirms the homogeneous distribution of ZnO NRs over a large surface area. The hexagonal shaped ZnO NRs are clearly seen in the high magnification image (Fig. 1b). NRs were observed to grow outward from the substrate with an excellent vertical alignment inline to the surface normal (Fig. 1c). The NR diameter vs. frequency was depicted in Fig. 1a (inset). The mean values of NR diameters and lengths are 0.6 µm and 48 µm, respectively constituting an ultra-high aspect ratio of ~ 80 (Fig. 1a inset). Figure 2 shows the XRD results confirming that the single crystalline ZnO NRs align along the c-axis (002), which could be due to the lowest surface energy attributed to the highly polar plane^40^. The hexagonal wurtzite crystal structure is very well matched with the standard diffraction pattern of the ZnO (JCPDS No. 36–1451)^33^. Figure 2 not only indicates the excellent crystalline quality of ZnO NRs as the XRD peak is very sharp and intense but also indicates the impurity free structures as they do not exhibit any other characteristic XRD peaks than those of ZnO. Figure 2 shows data for two types of ZnO NRs: ultra-long ZnO NRs (ZnO LNRs) synthesized in this work and relatively short ZnO NRs of ~ 9 µm (ZnO SNRs) exhibiting strong UV emission obtained elsewhere^41^. Shown in Fig. 2 (inset) are the magnified XRD peaks of ZnO NRs along with FWHM data. They both represent identical patterns with a change in intensity which is expected as longer NRs result in higher intensity.

**Figure 3:**
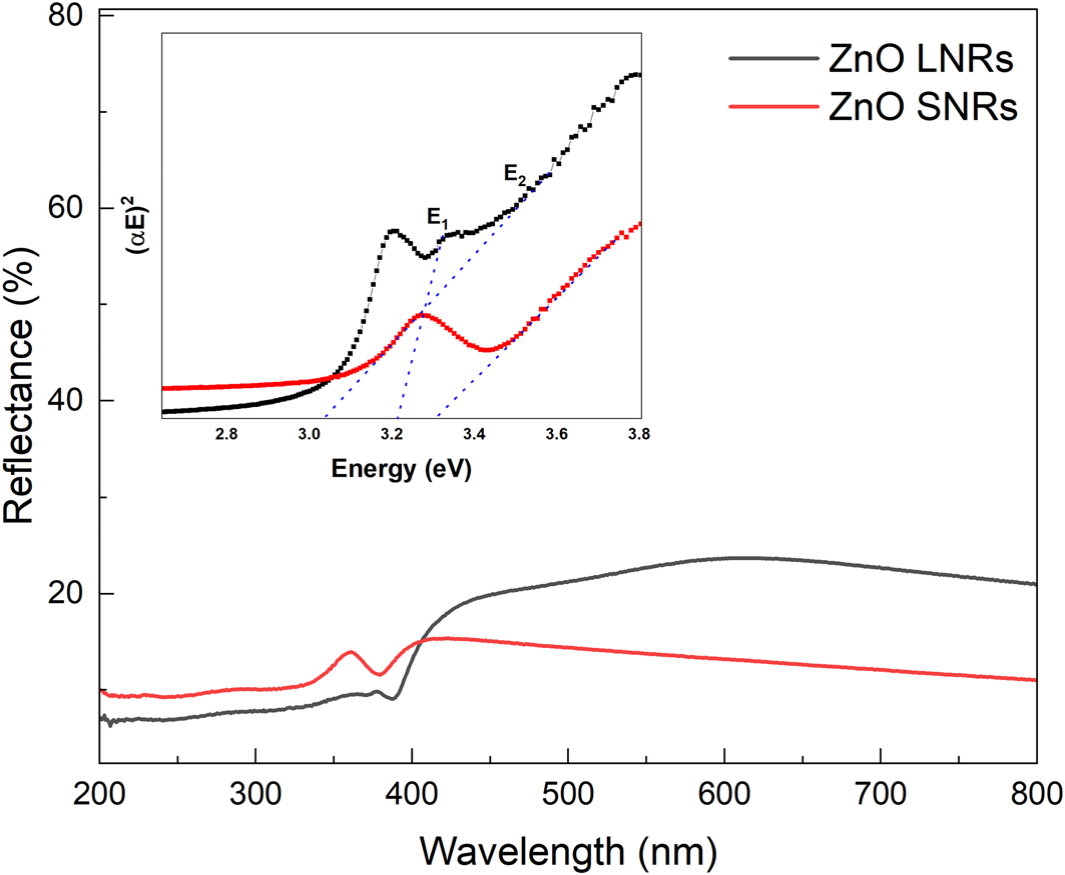
Reflectance analysis of the ZnO NRs. Tauc plot of the ZnO NRs (inset) two slope shift is observed in ZnO LNRs which refers to the high VE when compared to the NBE emission.

**Figure 4:**
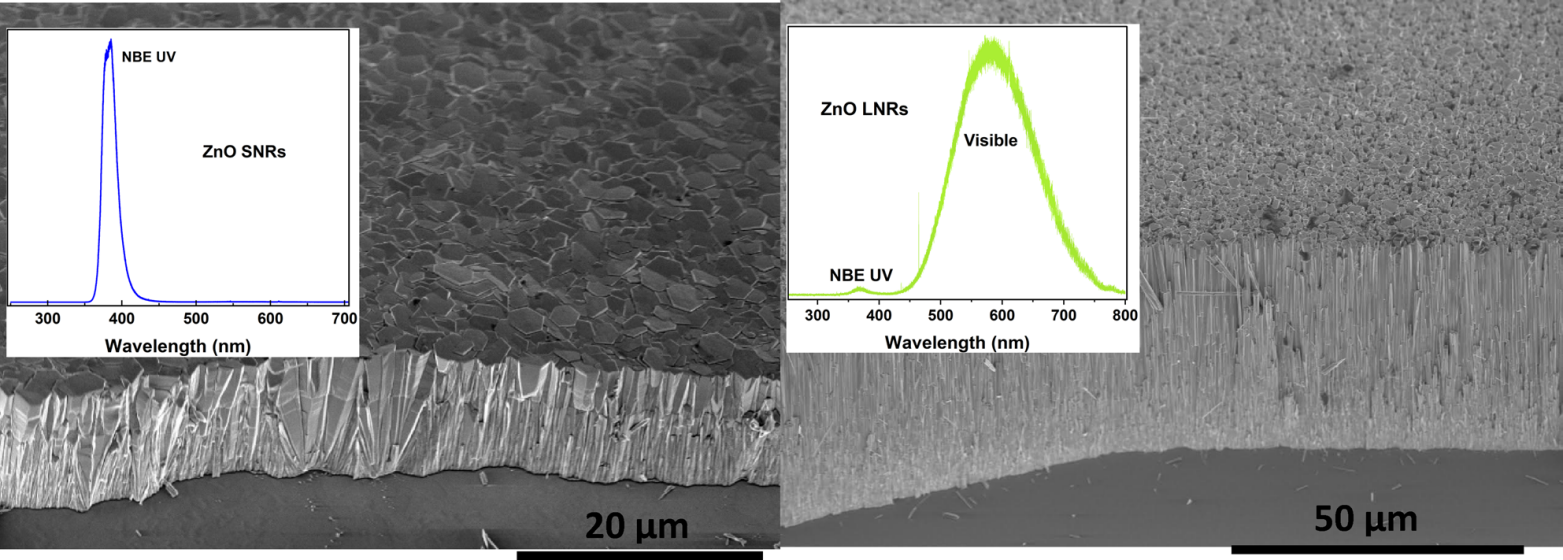
ZnO NRs with typical PL emission: ZnO SNRs with NBE UV emission (left), ZnO LNRs with VE (right).

Figure 3 shows the typical reflectance spectra of the ZnO NRs. The diffuse reflectance spectra represent over all antireflection with slightly higher reflectance but less than 30% in the visible region. Shown inset in Fig. 3 are the optical bandgaps of the ZnO NRs derived from Tauc plots. The bandgap was found to be 3.32 eV for ZnO SNRs. The bandgaps were 3.04, and 3.24 eV for ZnO LNRs. While these bandgaps lie within the standard range of a bulk ZnO crystal, the ZnO LNRs exhibit two band gaps corresponding to the direct (3.24 eV) and vacancy/interstitial (3.04 eV) associated bands in ZnO^42^. It has been reported that bigger size nanostructures displaying hexagonal ordering and long ranged periodic behavior, show enhanced photo-response and reduced bandgap which could be promoted by the higher concentration of oxygen vacancies present on these nanostructures^43^. Our results clearly confirm this state as the ZnO SNRs (smaller NRs) exhibit nearly pure near band edge (NBE) emission and represent a single band gap value while ZnO LNRs (bigger NRs) show very strong VE which could be originated from oxygen related defects and represent two absorption bands (Fig. 4). This red shift in the ZnO bandgap due to VE was reported to increase as the thickness of the material increases^44^.

**Figure 5:**
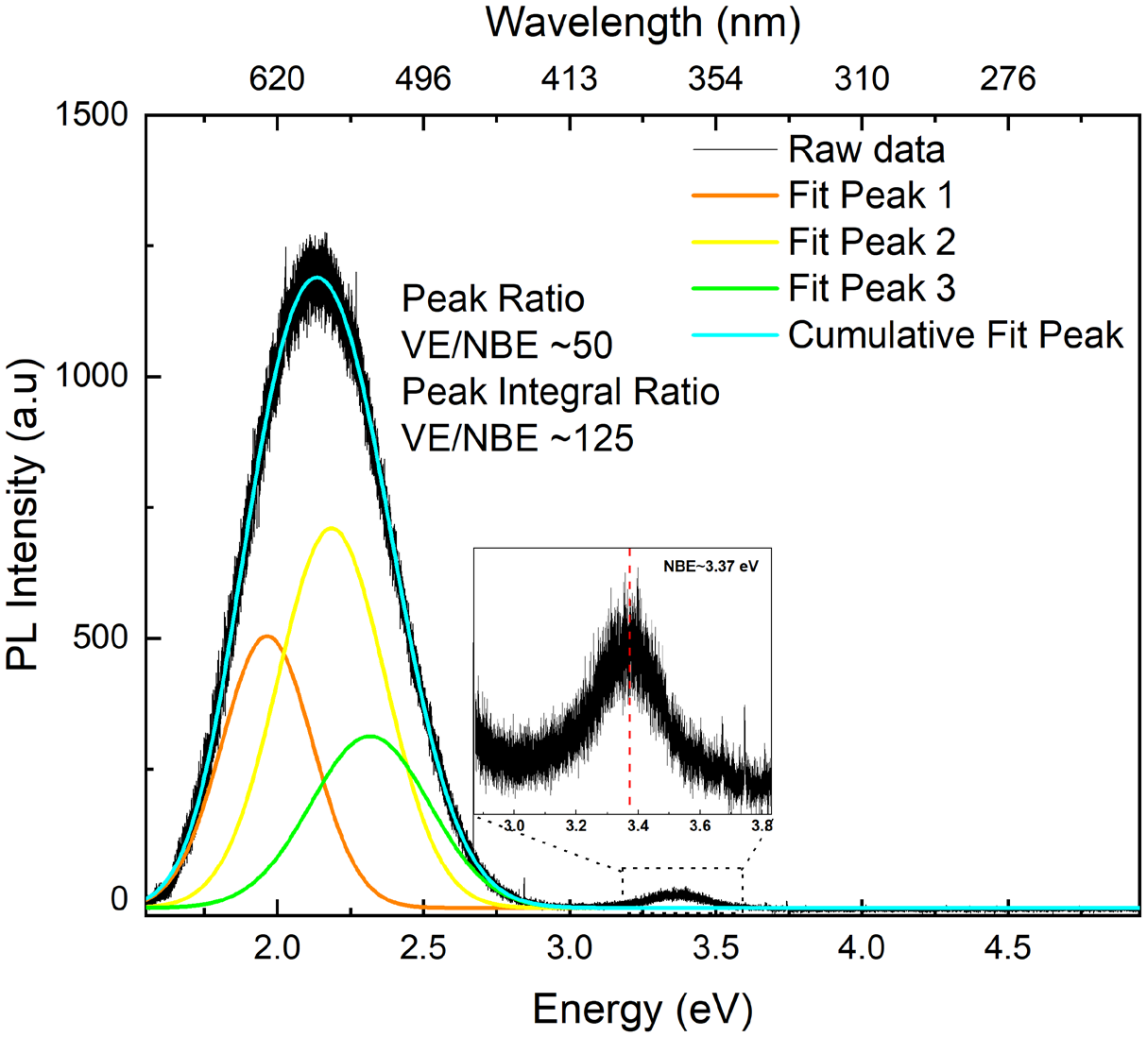
Room temperature PL spectra of the NBE and VE in ZnO NRs.

Photoluminescence measurements were performed for the optical properties and defect analysis. Ultra-long ZnO NRs show a broad and strong VE along with a weak NBE UV emission originated from exciton recombination (Fig. 5). The PL peak and peak integral ratios of VE/NBE were found to be ~ 50 and ~ 125, respectively. The excitation source was a He–Cd laser emitting 325 nm wavelength. Defect emission centered around ~ 580 nm (yellow) is in the visible region (450–700 nm) and the deconvolution of the spectrum covers mainly the emissions of green, yellow and orange-red emission bands (Fig. 5). These emissions are referred to the oxygen vacancy (V_O_) and interstitial (O_i_) related defect emissions. The green, yellow–orange and, red emissions are ascribed to the transitions from conduction band to the oxygen vacancy state, from conduction band to the oxygen interstitial state, and from Zn interstitial state to the oxygen interstitial state, respectively^42,45^. While recombination of electrons in singly (V_O_^+^) and doubly (V_O_^++^) charged oxygen vacancies with photo induced holes in the valence band was reported to result in green emission in ZnO^30,42,46^, recombination of electrons with deeply located holes in the oxygen interstitials (O_i_) was reported to result in yellow and orange emission^30,47^. The most prominent emission band is yellow as seen in Fig. 5. This intense yellow emission was seen in other studies including ZnO nanostructures and was attributed to the oxygen-vacancy related defects and recombination of the V_O_^++^ trapped center with delocalized electrons close to the conduction band as well^29,48,49^. It was also reported that the yellow emission is commonly observed in ZnO nanostructures prepared from aqueous solutions including zinc nitrate hydrate and HMTA and is attributed to oxygen interstitials (O_i_), Li impurities and Zn(OH)_2_ groups present in the structure^28,50–52^. While our results are in good agreement with the above statement as our samples are prepared from aqueous solutions including zinc nitrate hydrate and HMTA as precursors, it follows from the above discussions that the origin of the visible emissions is still under debate.

**Figure 6:**
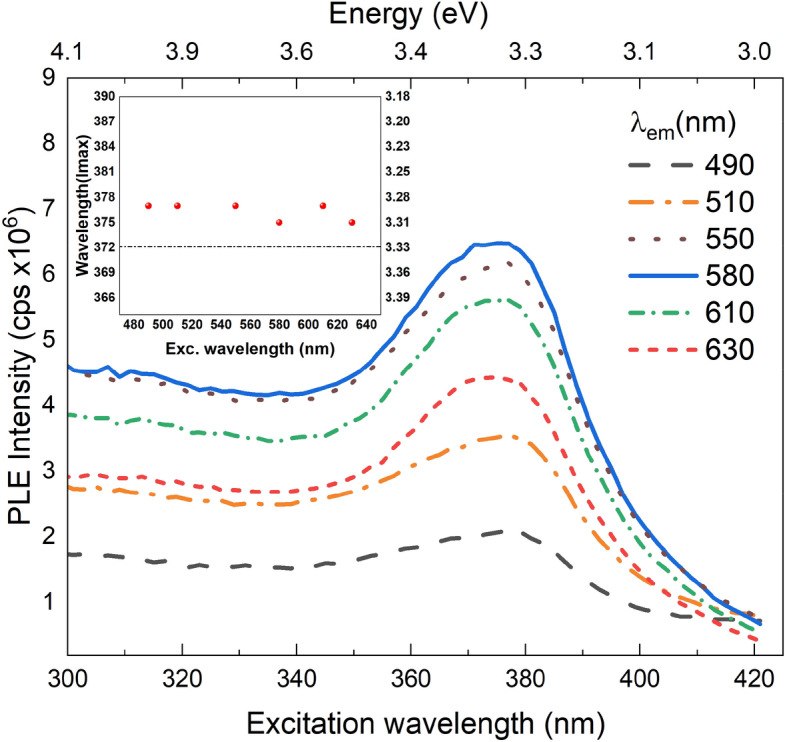
Room temperature PLE spectra of the ZnO NRs at different emission wavelengths. Shown in the inset is the wavelength at maximum peak intensity vs. excitation wavelength.

**Figure 7:**
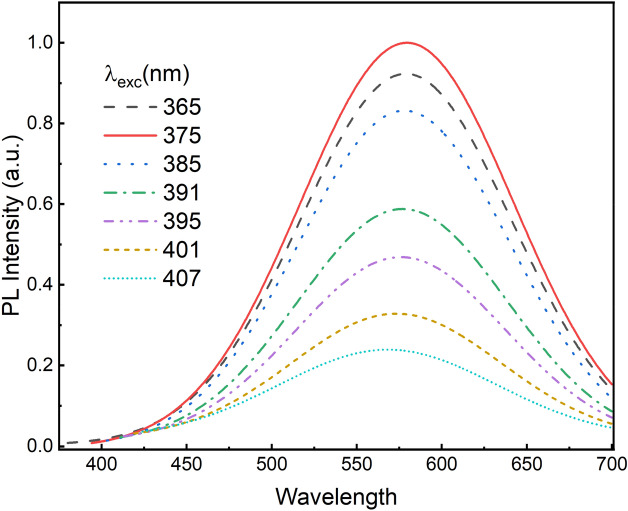
Room temperature PL spectra of the ZnO NRs at different excitation wavelengths.

**Figure 8:**
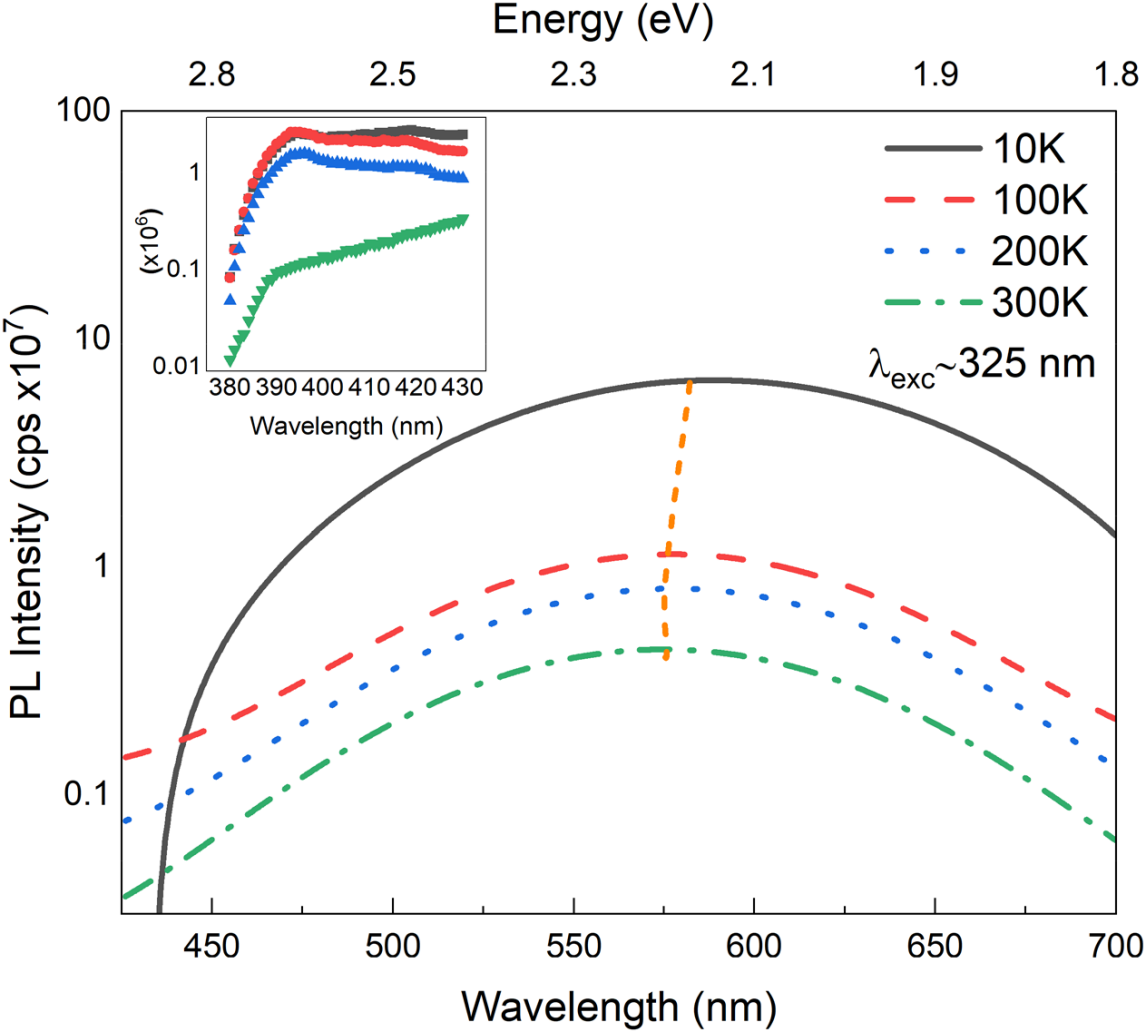
Temperature dependent PL spectra of the ZnO NRs. Inset shows the intensity of NBE emission as a function of temperature.

To better understand the VE related excited states, the photoluminescence excitation (PLE) measurements were conducted using a Xe lamp and dual scanning monochromators as excitation source. The typical PLE spectra of ZnO NRs is shown in Fig. 6 at various emission wavelengths. The dominant excitation peak has a mean wavelength in the range 375–377 nm as shown in inset Fig. 6. The highest emission intensity was observed for the yellow emission wavelength (~ 580 nm) as expected. Room temperature PL spectra at different excitation wavelengths are depicted in Fig. 7 indicating the same peak positions except for a gradual decrease in intensity after the excitation wavelength of 375 nm. Figure 8 shows the temperature dependent PL spectra for the ZnO NRs. It is seen that the intensity of the VE consisting of the green, yellow–orange and, red increases as the temperature decreases due to the freezing-out of phonons and nonradiative recombination quenching^51^. The VE peaks were shifted to the lower wavelengths as the temperature increased (Fig. 8). Figure 8 inset shows the NBE emission variation with the temperature which are significantly weak when compared to the VE. The intensity of the NBE emission was found to decrease with increasing temperature and results with quenching at room temperature. The decrease of excitonic emission intensity as the temperature increases might be related to the dissociation of the bound excitons due to the increased thermal energy. Also, the density of free carriers can be increased when the electrons trapped by native defects and impurities are released besides the free carriers excited from the energy band^49^. This will also reduce the surface depletion layer^53^ thickness but will increase the number of photo-generated holes at the surface. Surface adsorbed oxygen ions will therefore capture more holes leading to non-radiative recombination of captured holes with electrons via surface states and this will reduce the number of holes to participate in the excitonic transitions at high temperatures as well^49,54^.

**Figure 9:**
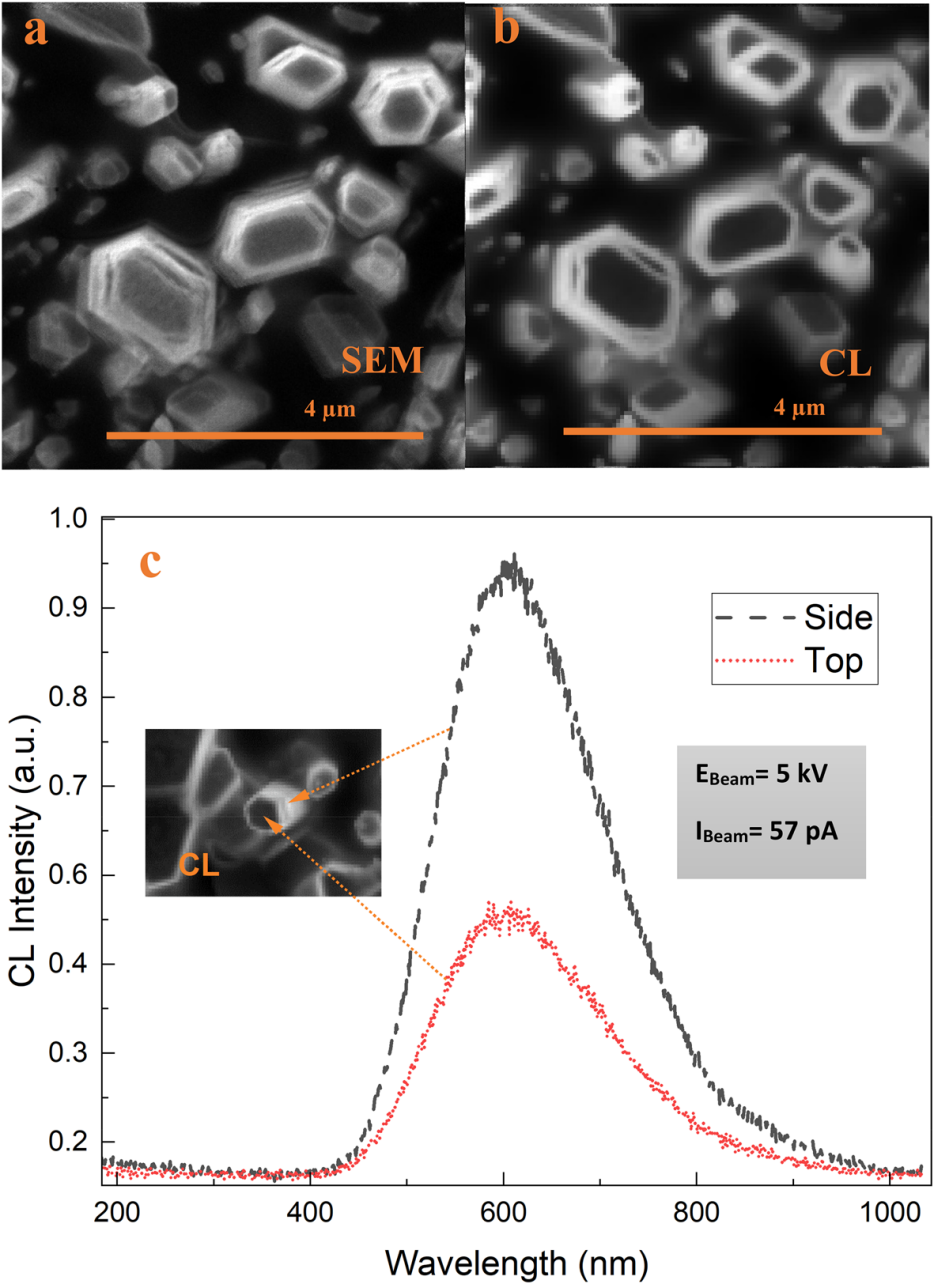
(**a**,**b**) SEM and CL images of the ZnO NRs (**c**) comparison of the CL spectra taken at the top and side region points of the ZnO NR.

**Figure 10:**
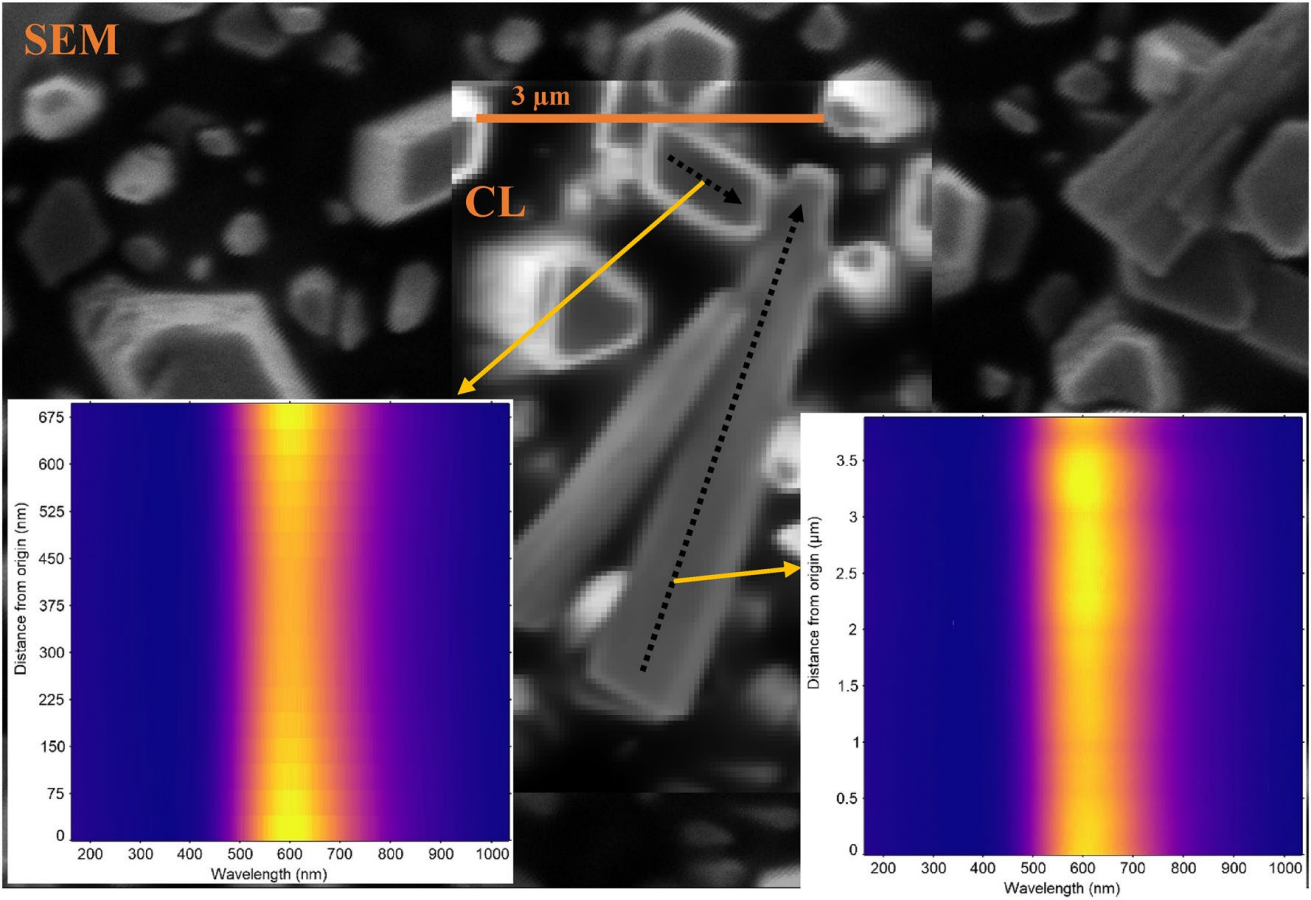
Comparison of the CL line scan for individual NRs and the top surface.

**Figure 11:**
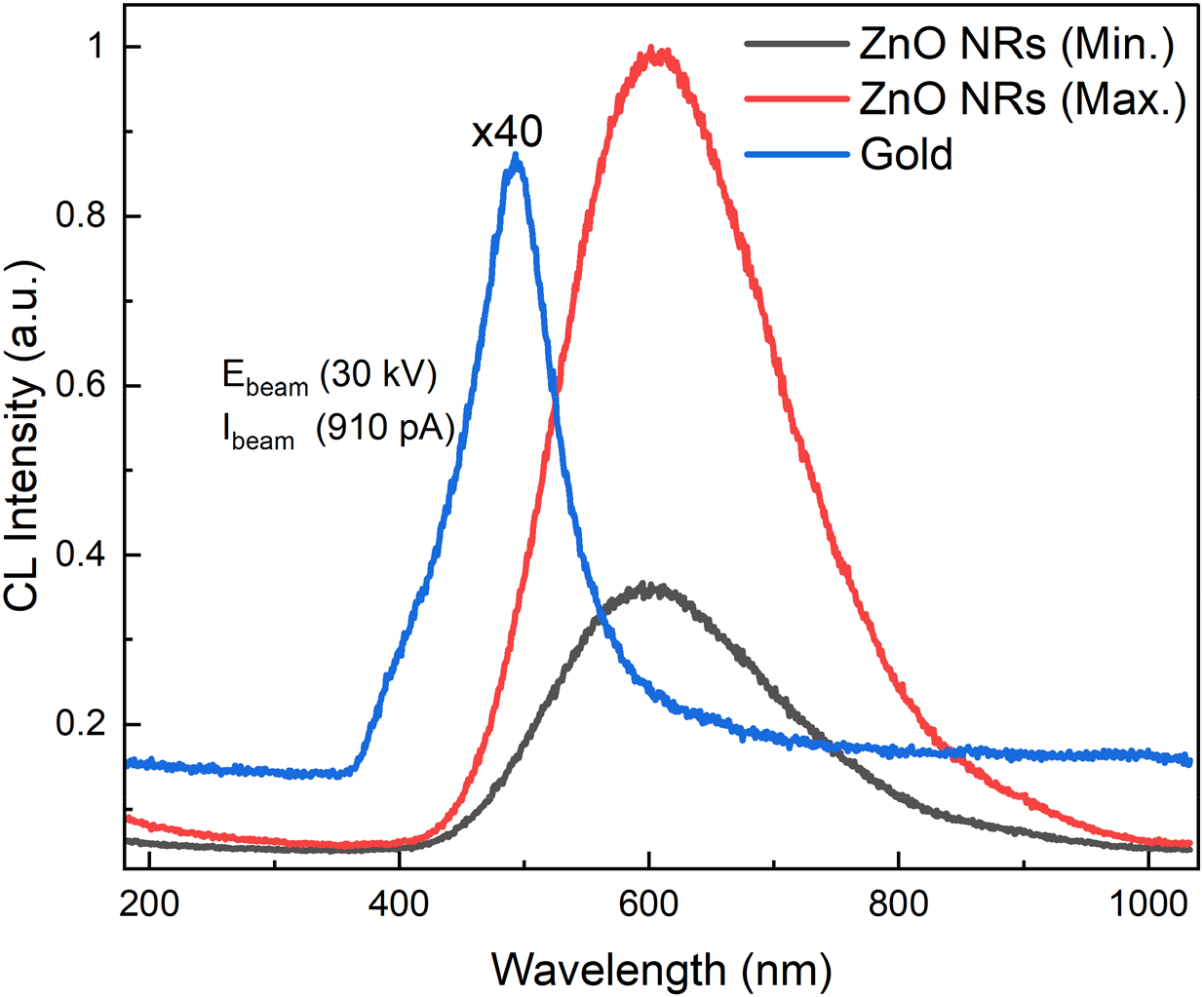
Comparison of the CL intensity of ZnO LNRs with TRCL of a gold calibration sample. TRCL of gold is magnified (×40) for convenience.

In order to investigate the spatial distribution of this VE, CL measurements were performed. In CL spectral imaging, a nanometer-scale converged electron beam excites the sample, and the luminescence subsequently emitted by the sample is collected by a high-numerical-aperture objective, hence it allows characterization of individual nanostructures with local non-uniform optical properties^55^. Figure 9a, b illustrates an SEM image along with the corresponding CL image (centered at ~ 600 nm with 146 nm bandwidth) mapping the spatial distribution of the VE intensity. As seen from the bright regions on the ZnO NRs in the CL ROI map, the VE originates from the lateral surface and near-edge top surface of the NRs. The CL emission intensity becomes significantly higher in the cross section of the NR than that on the top surface (Fig. 9c). Similar results were reported previously for the VE observation in ZnO NRs^55–57^. Figure 10 displays line scan profiles of the top surface and the cross-sectional view of an individual NR. It is seen from the line scan profile of the top surface of the NR that the CL VE is getting brighter near the edges of the surface and is quenched at the core of the NR (Fig. 10, inset left). Studies including the first principles density functional calculations of the defect formation energies by modelling polar and non-polar surfaces of the ZnO have shown that the O-vacancy related defect formation energy is lower near the boundary thus the VE is higher near the NR edges^58^. Therefore, the high density of radiative defects in the near-edge region of ZnO NR surfaces might cause the spatial variations in VE intensity^55^. The variation in the spatial distribution of VE across the cross section of an individual NR is shown in Fig. 10 (Inset, right) which confirms a slight increase in the VE intensity near the tip of the NR. It follows from above that the VE is more pronounced at the lateral non-polar surface (1010) rather than the top polar surface (0001), which is very well defined in XRD patterns (Fig. 2). We found that all the ZnO nanorods investigated are luminescent as the CL images represent clearly visible NRs as they appear in the corresponding SEM images. The ZnO NR CL at the side (Max.) and the top (Min.) of the NRs is substantially brighter than the transition radiation CL (TRCL) generated by a gold calibration sample (100 nm Au on 5 nm Cr adhesion layer on Si) with a 30 kV excitation as shown in Fig. 11, despite the lower beam peak voltage (5 kV) used for the ZnO CL measurements. The 30 kV beam peak voltage was used for the gold transition radiation measurements because the transition radiation CL amplitude was immeasurably small at 5 kV. A comparison between 5 and 30 kV CL spectra acquired from ZnO SNRs is shown in supplementary information Fig. S1 online.

**Figure 12:**
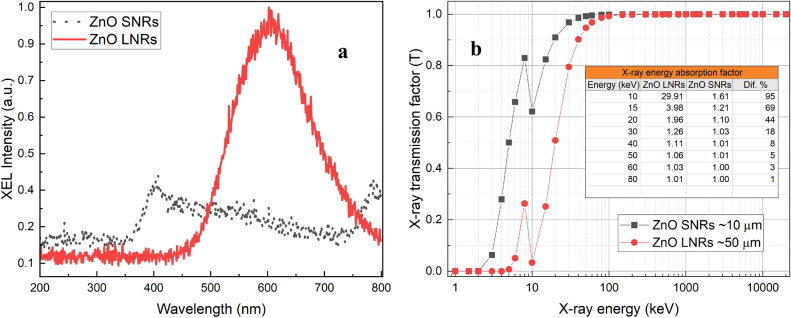
(**a**) XEL emission spectra for the ZnO NRs (**b**) X-ray transmission and energy absorption in ZnO NRs.

Besides the PL and CL measurements, the X-ray excited luminescence (XEL) is a key measurement for evaluation of materials to be used as scintillators for high quality X-ray detection and imaging^9^. It constitutes a basis for scintillator material assessment in the field of ultrafast and hard X-ray detection^6^. While the room temperature PL is dominated by impurities or surface defects, XEL is dominated by internal indirect luminescence as X-rays are highly penetrative into the structure. It is the result of different energy-transfer processes including core–hole decay and cascade dynamics, and is also a highly material dependent process sensitive to composition, structure, morphology, size, crystallization and defects, etc.^10,59,60^ Fig. 12a shows the XEL spectra of the ZnO NRs in the range of 200–800 nm. The effective density and irradiated area of ZnO NRs were the same during measurements. A significantly higher intensity XEL emission peak in the visible region is observed in ZnO LNRs when compared to the ZnO SNRs (emitting a strong NBE and a weak VE) indicating a promising scintillation application of ZnO LNRs in X-ray imaging. The higher light output observed from the ZnO LNRs can be attributed to several factors. Increased self-absorption might cause the NBE in ZnO SNRs to be less efficient compare to VE in ZnO LNRs. The XEL peak emission centered around 600 nm also matches well with the diameters of the ZnO LNRs as seen in Fig. 1a. This could result in greater optical coupling and light extraction efficiency. Furthermore, the ZnO LNRs have more than five times greater thickness than the ZnO SNRs, with a resulting increase in X-ray attenuation. Figure 12b represents the X-ray interaction in both ZnO LNRs and ZnO SNRs. The X-ray transmission factors (I/I_0_) were calculated based on the Beer–Lambert law using the known values of X-ray mass energy absorption coefficients^39^, thickness and density data and are depicted in Fig. 12b. It is seen that the X-ray transmission is less in ZnO LNRs at energies below 80 keV due to the greater material thickness. Figure 12b inset shows the linear energy absorption factors (I_0_/I) indicating that the ZnO LNRs have significantly higher energy absorption than the ZnO SNRs resulting in higher XEL intensity for X-rays ≤ 30 keV and for X-ray energies greater than 30 keV, the X-ray absorption efficiency of ZnO LNRs is not significantly higher than that of ZnO SNRs. Therefore, it is essential to maximize the X-ray energy deposition in the scintillator material in addition for the need to produce a highly luminescent material for a high spatial resolution detector application. A comparison has been made between the scintillation brightness of the ZnO NRs and a standard BGO scintillator (Supplementary information Fig. S2 online). However it is not a direct comparison of performance since the BGO sample is significantly thicker than the ZnO NRs. At 30 keV, a 4 mm thick BGO fully absorbs the X-rays (mean free path ~ 60 µm) compared to 50 µm thick ZnO nanorods which absorb ~ 30% of the incident X-rays (mean free path ~ 180 µm) (Supplementary information Fig. S3 online).

**Figure 13:**
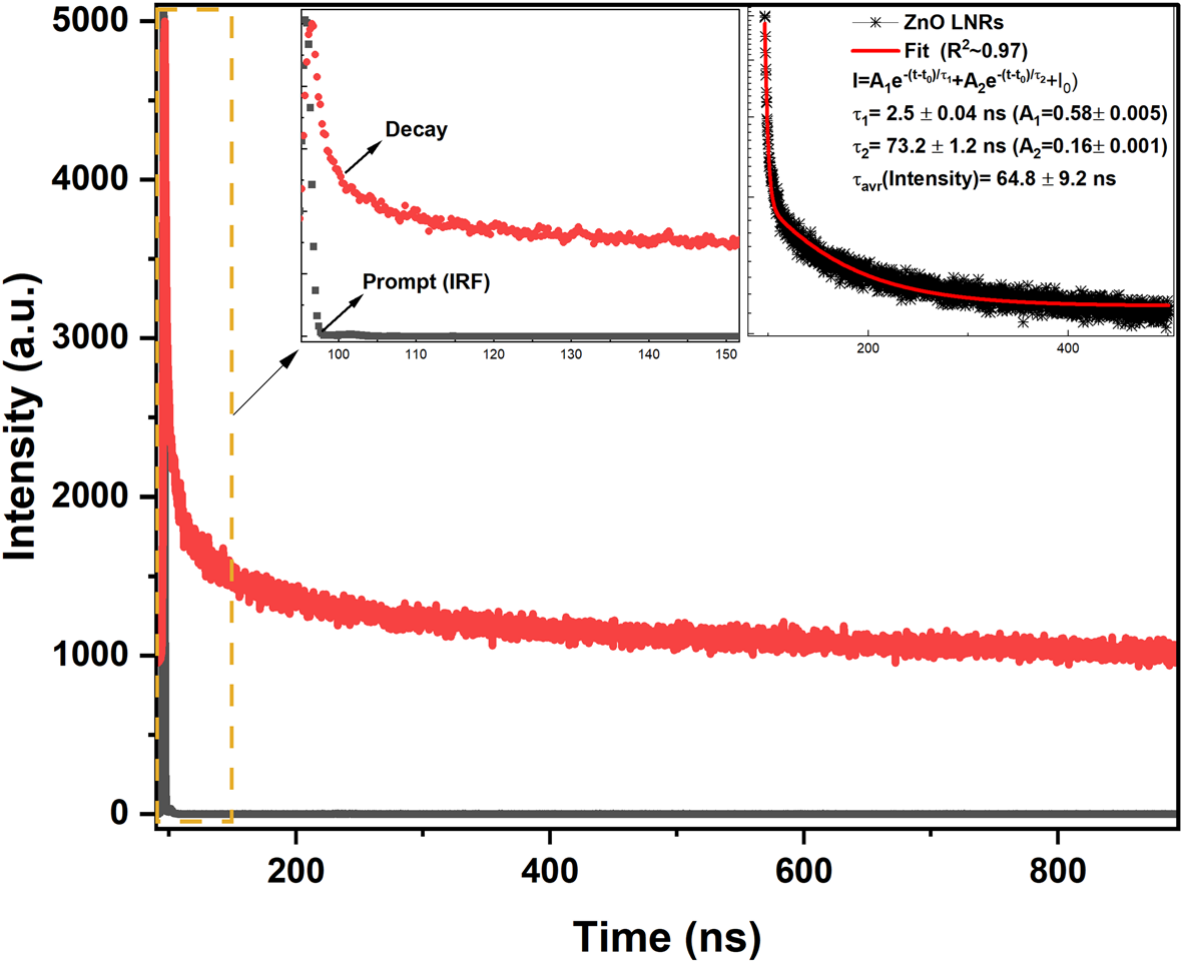
Time resolved PL spectra including decay profile of the ZnO LNRs at 580 nm.

In order to meet demands for fast X-ray imaging detectors, the scintillator screens must have fast decay time properties. Although the NBE UV emission in ZnO is known to have superfast sub-nanosecond ultraviolet luminescence, the VE still satisfies the condition of a fast X-ray detector with its nanosecond-scale decay time^35,36,61^. The room temperature time resolved PL (TRPL) technique was used to determine the decay time response. Figure 13 shows the obtained decay curve of the ZnO NRs at 580 nm. The measured TRPL spectra is well fitted to a two exponential fitting function and the time decay constants were found as τ_1_ = 2.5 ± 0.04 ns, τ_2_ = 73.2 ± 1.2 ns constituting the fast and slow components, respectively. As seen from Fig. 13, the fast decay component of around a few ns and the average decay times in the ns scale confirms promising properties of ZnO LNRs to serve as a fast scintillator in ultra-fast high-resolution X-ray detector applications. Studies are in progress to obtain doped ZnO NRs with greater length and enhanced structural and optical properties.

## Conclusions

In the present research, high aspect ratio well-aligned nanostructured ZnO scintillators were grown for fast X-ray detection applications. These scintillators are based on a vertically aligned nanorod array design increasing the effective surface area and efficiency of the scintillator instead of the planar scintillator design i.e. bulk crystal and thin films. A cost-effective low temperature hydrothermal method was adopted to grow vertically aligned ultra-long (~ 50 µm) ZnO nanoarrays in one cycle growth and their structural, optical and X-ray scintillation properties were investigated. ZnO NRs have shown high crystal quality under SEM and XRD characterization and have resulted in efficient luminescence intensity in the visible region under PL, CL and XEL characterization. CL measurements revealed that the VE intensity is significantly high at the lateral non-polar surface (1010) and near edge top surface and is more quenched at the top polar surface (0001). From the optical measurement results, it has been shown that the ZnO NRs are luminescent and their vertically aligned nanoarray design helped optical coupling and thus enhanced light extraction. Further improvement in decay time responses and the XEL and CL measurement results could indicate promising properties of these materials for use in ultra-fast high-resolution X-ray detectors.

As a summarize, the growth and characterization of thick (50 microns) ZnO nano scintillator arrays were reported in this work. While the growth of ZnO NRs is already well known in the literature, the maximum reported thickness for the ZnO nano scintillators is generally ~ 10–20 micron. Here we report the growth of thicker, very well-defined, and hexagonal ZnO NR films, with very high aspect ratio and emitting in the visible range, which could find application in fast X-ray imaging. Moreover, the defect engineering is also described with oxygen vacancy related defects, to tune the emission in the visible region of the optical spectrum.

## Methods

ZnO nanorods (NRs) were synthesized by the low temperature hydrothermal technique on silica glass substrates. A deionized (DI) water solution containing precursors such as zinc nitrate (Zn(NO_3_)_2_.6H_2_O, Sigma Aldrich, 98%) and hexamethylenetetramine (HMTA, C_6_H_12_N_4_, Sigma Aldrich, ≥ 99%) was prepared first with a molar ratio of 2:1. Then, 0.8 M ammonium hydroxide (NH_4_OH, Sigma Aldrich, ACS reagent) and 5 mM polyethylenemine (PEI, (C_2_H_5_N)_n_, Sigma Aldrich) were added in the solution to further control the ZnO NR growth. A 100 nm thick ZnO seed layer pre-coated substrate was immersed in the solution for 25 h at 95 °C. ZnO seed layer was sputtered on the substrate using an RF sputtering equipment with Ar ion bombardment of the target. Following growth of ZnO NRs, samples were thoroughly washed with DI water to remove any residual salts. The kinetics of growth and chemical reactions were explained in detail elsewhere^31^. Briefly, to initiate the growth of ZnO NRs the zinc salt hydrolyze into a supersaturated solution. The precursors zinc nitrate and HMTA supply zinc and hydroxyl ions, respectively. The latter one is a buffer like component maintaining ZnO growth continuously as the hydroxyl ions slowly release into the solution. When ammonium hydroxide is used as an additive, it immediately coordinates with zinc ions thereby lowers the concentration of free zinc ions and the precipitation in the solution. Finally, PEI is used as another additive in the solution to further control the growth of the ZnO NRs. It attaches to the lateral nonpolar surfaces and hinders the lateral growth but enhance the longitudinal growth along the c-axis.

For characterization of structural properties a scanning electron microscope (HITACHI SU8230 SEM) was used to get tilted top view and cross sectional view of the ZnO NRs. It is equipped with a cold field emission gun facilitating relatively low voltage imaging with high resolution. An X-ray diffractometer (Panalytical XPert PRO, XRD) with a Cu as anode material (Κα = 1.54187 Å) was used to analyze crystal structure along with phase identification. The observed XRD peaks are in very well alignment with the indexed reflections of hexagonal wurtzite ZnO structure (JCPDS No. 36–1451). A He–Cd 325 nm UV laser (Kimmon Koha, Tokyo, Japan) and a PL spectrometer (Renishaw, Wotton-under-Edge, UK) were used for the room temperature PL measurements. Reflectance measurements were performed using a Cary 5000 UV/Vis/NIR spectrophotometer with tungsten halogen and deuterium arc light source (Agilent, Santa Clara, CA, USA).

Steady-state photoluminescence emission (PL) and excitation (PLE) were measured with a Horiba Jobin Yvon Fluorolog 3 spectrofluorometer equipped with a Xe lamp and dual scanning monochromators. A 320 nm optical filter was used for blocking the second order excitation wavelength. The PL lifetime was measured using a time-correlated, single photon counting technique. The excitation sources were NanoLEDs with 1 ns pulse widths, and emission wavelengths in the range of 265–700 nm were monitored. PL spectra were corrected for instrumental response/distortion.

For low-temperature measurements, samples were placed in an optical O-ring sample holder where the sample was held between two pieces of silver foil with a window from one side. The sample holder was attached to the cold finger of a DE202AE cryostat. The temperature was manually set using a Lakeshore 331 temperature controller. A 380 nm optical filter was used for blocking the second order excitation wavelength.

The radioluminescence (RL) measurements were done under continuous 30 keV X-ray irradiation using a CMX003 X-ray generator. The emission spectra were recorded in reflection geometry using a 150 mm focal length monochromator over a wavelength range of 200–800 nm.

The cathodoluminescence (CL) microscopy was performed in a FEI Quattro environmental SEM with a beam energy of 5 keV and a beam current of 14 and 57 pA. All measurements were performed in a 250 mTorr water vapor background to suppress charging. No sample degradation was observed after CL measurements using these beam conditions. A Delmic Sparc cathodoluminescence module was used to collect the light generated by the sample; a parabolic mirror with a numerical aperture of 0.97 collimated the light and directed it out of the SEM chamber, where it was focused onto the slit of an Andor Kymera 193 spectrograph and measured on an Andor Newton CCD camera.

### Supplementary Information

Supplementary Figures.

## Data Availability

The data that support the findings of this study are available from the corresponding author upon reasonable request.
