# Supplementary material for: Solution processed high aspect ratio ultra-long vertically well-aligned ZnO nano scintillators for potential X-ray imaging applications
Source: Sci Rep. 2024 Jul 9;14:15803. doi: 10.1038/s41598-024-61895-6 (PMC11233702; doi:10.1038/s41598-024-61895-6)
Supplement: Supplementary file 1 — Supplementary Figures. [file 41598_2024_61895_MOESM1_ESM.docx]

Supplementary information for “**Solution processed high aspect ratio ultra-long vertically well-aligned ZnO nano scintillators for potential X-ray imaging applications**”


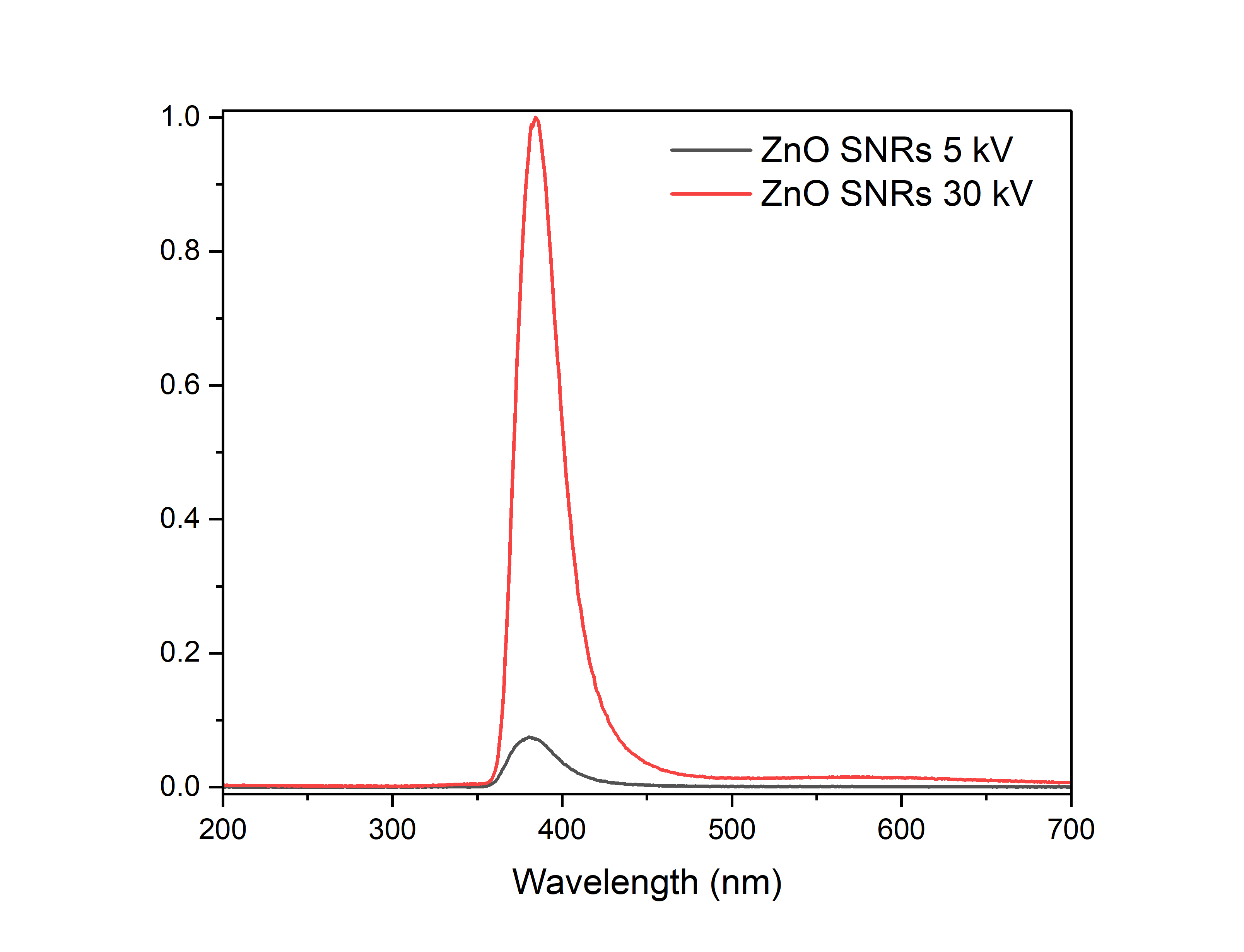


**Figure S1** CL intensity of ZnO SNRs emitting in the UV region, acquired with a X-ray tube voltage of 5kV and 30kV^[[1]](#footnote-1)^.


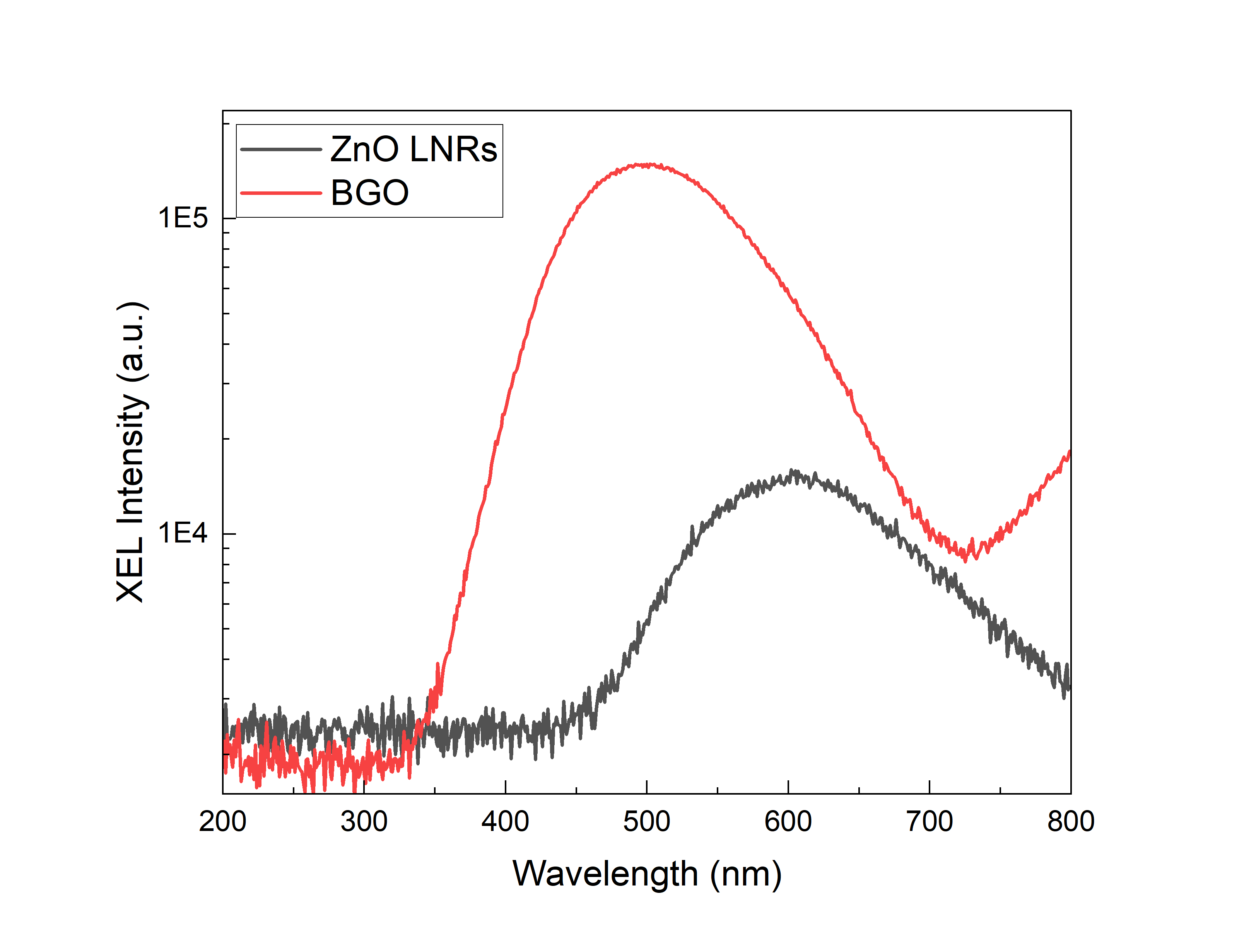


**Figure S2** Comparison of the XEL intensity of 4 mm thick BGO scintillator and the 50µm thick ZnO LNRs, with similar sample areas. The X-ray tube voltage was 30 kV.


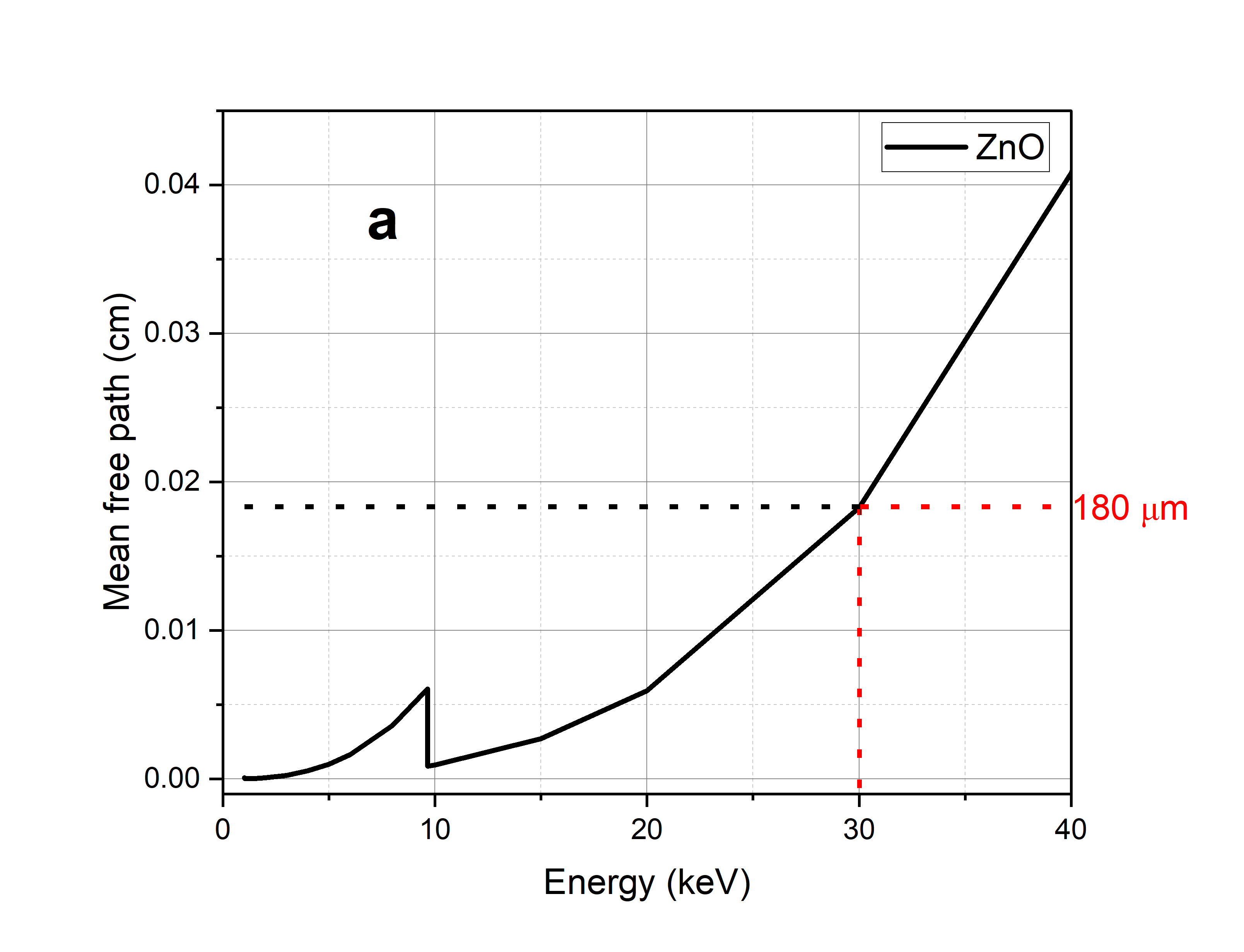

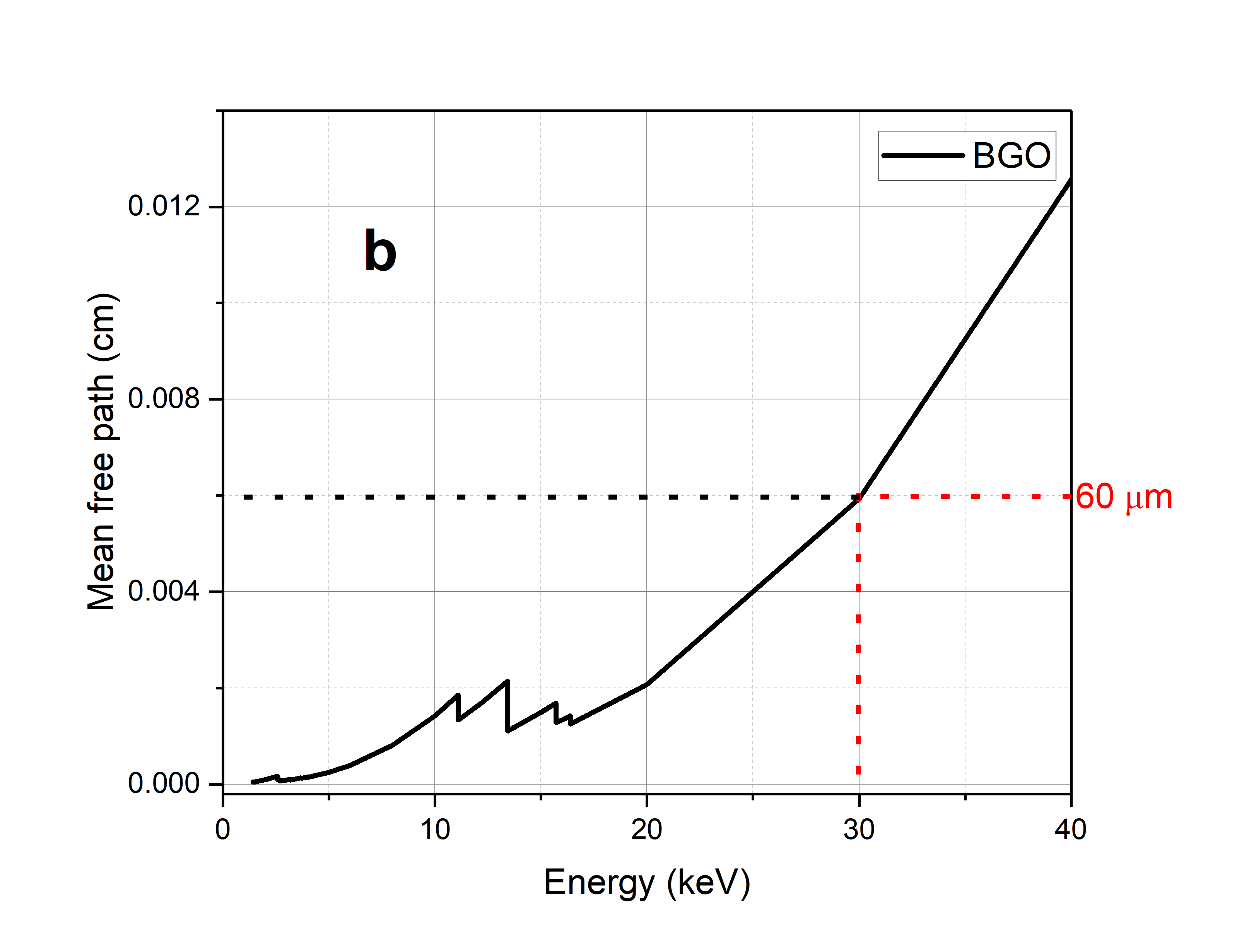


**Figure S3** (a,b) X-ray mean free paths of BGO and ZnO as a function of X-ray energy, calculated from mass attenuation coefficient data obtained from NIST XCOM.

1. The mean energy of the X-ray beam is about 2/3 of the maximum energy depending on the type of anode material, and any filtering used on the beam. [↑](#footnote-ref-1)
